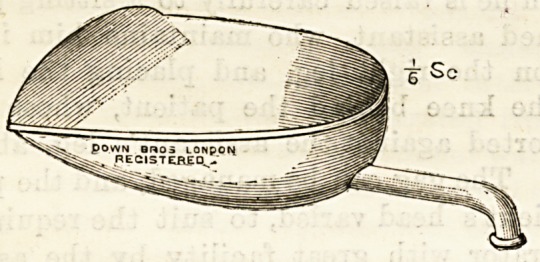# New Appliances and Things Medical

**Published:** 1895-10-12

**Authors:** 


### NEW APPLIANCES AND THINGS MEDICAL.

[We shall be glad to receive, at our Office, 428, Strand, London, W.C., from the manufacturers, specimens of all new preparations and appliances which may be brought out from time to time.]

### IMPROVED IRRIGATING TRAYS.

For dressing wounds which require prolonged irrigation with large quantities of fluid two new varieties of trays, of which one is here illustrated, will be found very convenient. They are flat, made of hammered copper, plated, and with a curved back to prevent the fluid escaping over the hand. The bent tube which serves as a handle and delivery tube combined is fixed as close to the tray bottom as possible. The front edge is made concave or convex in outline and bent somewhat upwards to ensure a surface as well as edge contact; the shape of the front edge and, of course, the size of the tray can be altered for special purposes, and the delivery tube can be made to

$\frac{1}{6}$ Sc telescopic or to screw on and off. Rubber tubing attached to the end of the hollow handle will convey the contents away into a pail or basin placed for its reception. The makers, Messrs. Down Brothers, St. Thomas's Street, E.C., have well carried out the suggestions of the originator of this appliance, which may certainly claim the practical advantages of helping to prevent unnecessary mess and spilling, and of avoiding a frequent change of receptacles, by allowing continuous irrigation. The tube is large enough to allow the passage of lymph flakes, blood-clots, &c., and "kinking" is prevented by the downward bend being on the metal portion of the handle. Nurses, we feel sure, will be glad to welcome this useful little invention.

### LACTOPEPTINE TABLETS.

(John M. Richards, 46, Holborn Viaduct, E.C.)

As one of the outcomes of the "new pharmacy," patients will welcome the introduction of lactopeptine tablets. So many drugs are now dispensed in this manner, that patients often ask for tablets in preference to powders or pills. Lactopeptine has long been familiar to dyspeptics in the first-mentioned form, and in spite of the inconvenience of swallowing a powder, and carrying it about in so unhandy a form, many persons have been in the habit of taking a few grains regularly after each meal. A man can now go out to dinner and take his lactopeptine with him, and not attract undue attention as he quietly swallows a ten-grain tablet.

## Figures and Tables

**Figure f1:**